# Novel Design of the Chimeric Deep Inferior Epigastric Artery Perforator Flap that Provides for Three‐Dimensional Reconstruction of Composite Tissue Defects of the Heel in Children

**DOI:** 10.1111/os.12887

**Published:** 2021-01-15

**Authors:** Junyi Yu, Zhenhua Luo, Panfeng Wu, Juyu Tang

**Affiliations:** ^1^ Department of Hand and Microsurgery, Department of Orthopaedics, Xiangya Hospital Central South University Changsha China

## Abstract

**Objective:**

The aim of the present study was to report a novel design of the chimeric deep inferior epigastric artery perforator flap (DIEP) to achieve dead space filling, Achilles tendon bridging, and skin resurfacing simultaneously with minimal donor‐site morbidity.

**Methods:**

From September 2012 to May 2016, a retrospective study was carried out on six pediatric patients with composite soft tissue defects of the heel that were repaired with the chimeric DIEP flap. The chimeric flap design included a flap of the anterior sheath of the rectus, a block of rectus muscle, and a large skin paddle. All the parts were supplied by a common artery. After harvesting the flap, all element parts were inserted at the corresponding sites in a tension‐free manner. With one set of vessel anastomoses at the recipient site, accurate repair with tendon reconstruction, dead space elimination, and wound covering were accomplished. The donor site incisions were closed initially. Data on patient age, medical history, injury severity, defect size, flap dimensions, recipient vessels, donor site closure, complications, and follow‐up were collected and reviewed.

**Results:**

Five of the six chimeric DIEP flaps survived without complications. The remaining one case experienced partial necrosis of the skin paddle caused by venous congestion, which healed after routine dressing changes. Primary donor site closure was accomplished in all cases. The mean follow‐up was 18.6 months (range, 10–36 months). Five patients had satisfactory aesthetic and functional outcomes; one patient needed a secondary debulking procedure. Compared to the unaffected side, the affected side showed no obvious difference for ankle movement, tiptoe function, and patient gait during the follow‐up period. Good ankle function was observed in all patients. There was no donor site breakdown, with only a slightly noticeable linear scar.

**Conclusion:**

The chimeric DIEP flap reduced the operative time, solved the problem of deficiency of recipient vessels, and attained satisfactory functional and aesthetic outcomes with low donor site morbidity. Therefore, it is a promising option for three‐dimensional reconstruction of composite defects with dead space and Achilles tendon defects as well as skin loss in children.

## Graphical abstract

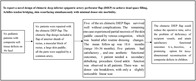

We reported a novel design of the chimeric deep inferior epigastric artery perforator flap (DIEP). The chimeric DIEP flap reduced the operative time, solved the problem of the deficiency of recipient vessels, and attained satisfactory functional outcomes. Therefore, it could be a promising option for three‐dimensional reconstruction of composite defects in children.

## Introduction

Heel composite soft tissue damage in children, including loss of skin concomitant with dead space and Achilles tendon defects caused by trauma, remains a huge challenge for reconstructive surgeons. The pathologic features of composite defects require reconstruction to meet the demands of covering, bridging, and filling the heel simultaneously at the least cost^1^, ^2^. Efforts have been made in various medical centers to repair similar multiplanar defects in adults, including the use of local cutaneous flaps, pedicled fasciocutaneous flaps, and microsurgical free flaps^3^, ^4^. However, these procedures are less applicable in pediatric patients due to the limited amount and variety of donor tissue and the small vessel caliber. In addition, the huge unsightly scar on the crus affects pediatric patients physiologically and psychologically^5^, ^6^. Some published studies report adding one or two independent free vascularized flaps based on the conventional design^4^, ^7^; the approach required more effort in flap harvesting as well as more recipient vessels, which prolonged surgery and increased the morbidity in both donor and recipient sites. The physiological characteristics of children and the concerns about future growth and development make the equilibrium between wound covering and donor site compromise difficult to achieve^1^, ^3^.

Recently, numerous pioneering studies have been published in which the perforator‐based chimeric flaps were applied for economical tissue use^8^, ^9^, ^10^, ^11^. The chimeric flap has separate components with independent vascular supplies attached to a common pedicle, thus reducing the operative time and overcoming the obstacle of deficiency of recipient vessels. The deep inferior epigastric artery perforator (DIEP) technique has gained great popularity in breast reconstruction and the repair of defects in other parts of the body, including the trunk and extremities^1^, ^12^. Based on the demonstrated feasibility and effects in former work, the combination of the chimeric design and the DIEP flap would be an ideal option in pediatrics because of the abundance and variety of tissues that can be used with this approach. This chimeric DIEP flap procedure requires a skin paddle, a fascia flap of the anterior sheath of the rectus, and a block of rectus muscle for wound coverage and involves Achilles tendon reconstruction and elimination of dead space, with minimal donor site compromise. Without the need for frequent changes in body position and donor site, it reduces efforts in flap harvesting and grafting, thus lowering the surgical risks, leaving less conspicuous scars, and only minimally affecting growth and development. Although there are a limited number of studies focusing on the reconstruction of composite defects in the head and neck using the chimeric DIEP flap^13^, few scholars extend its application to limb repair. In this case series, we present our experience using a free chimeric DIEP flap in a single procedure to rebuild the heel after severe injury for function and aesthetics. This chimeric design consists of a skin paddle, a fascia flap of the anterior sheath of the rectus, and a block of rectus muscle. The procedure fulfills the requirements of covering, bridging, and filling. We aim to (i) functionally and aesthetically repair the heel following severe injury in children; (ii) assess the feasibility and reliability of this new design; and (iii) provide the basis for further study.

## Methods

### *Patients*

From September 2012 to May 2016, six pediatric patients, ranging in age from 6 to 11 years, received free chimeric DIEP flaps for reconstruction of soft tissue defects in the heel. For the present study, demographic data, intraoperative data, early complications data, and long‐term follow‐up results were collected.

The main cause of defects is trauma, including motorcycle spoke and crushing injuries. Initial debridement was carried out at nearby hospitals before the patients were transferred to our center. This study has been approved by the ethics committee of our hospital. Signed informed consent was obtained from parents or guardians of the children. Detailed information on each patient is provided in Table 1.

**TABLE 1:** Patient list

Case	Age/Sex	Defect location	Defect of layers	Cause	Type of compound flap	Recipient vessels	Dimension of skin (cm^2^)	Dimension of muscle and fascia (cm^2^)	Donor site closure	Complications	Follow up (months)
1	8 year/M	Right heel	All layers	Motorcycle spoke injury	Type 2	Posterior tibial artery	12 × 6	5 × 3 4 × 2	Primary	None	24
2	11 year/M	Right heel	All layers	Ulcer	Type 1	Anterior tibial artery	10 × 5	7 × 2 5 × 2	Primary	None	14
3	6 year/M	Right heel	All layers	Motorcycle spoke injury	Type 1	Posterior tibial artery	12 × 6	6 × 3 5 × 2	Primary	Partial flap necrosis	36
4	7 year/M	Right heel	All layers	Motorcycle spoke injury	Type 2	Posterior tibial artery	13 × 4.5	9 × 3 4.5 × 2	Primary	None	10
5	8 year/M	Left heel	All layers	Motorcycle spoke injury	Type 2	Posterior tibial artery	9 × 5	4 × 2 5 × 2	Primary	None	18
6	7 year/M	Right heel	All layers	Motorcycle spoke injury	Type 2	Posterior tibial artery	11 × 5	6 × 3 4 × 2	Primary	None	10

M, male.

The inclusion criteria were^1^: patients younger than 14 years of age^2^; patients suffering from trauma‐caused soft composite defects in the heel^3^; patients with no serious underlying disease^4^; and the parents and guardians of the patients agreed to undergo one‐stage reconstructive surgery with a free chimeric DIEP flap. Exclusion criteria were patients: (i) with soft tissue defects resulting from the removal of a tumor in the heel; and (ii) with deep inferior epigastric artery dysplasia.

### *Operative Technique*

#### *Preoperative Preparations and Flap Designs*

Routine ultrasonography of lower limb vessels was performed to check the continuity of the main arterial trunk of the involved heel and to make sure that no anatomical variations or underlying vascular disease existed.

General anesthesia was performed, and the patient was placed in prone position. We first assessed the size of the defect, the length of the Achilles tendon defects, and the volume of the dead space after a thorough debridement. We then drew the contour of the skin defect on a polyethylene sheet and a tailored sheet as a template.

The available perforators were detected and marked on the patient's abdominal wall with the help of a hand‐held Doppler unit. Based on the perforator positions and the size and three‐dimensional features of the defects, we marked the design (location, size, and orientation) of each tissue paddle.

#### *Approach and Pedicle Dissection*

A skin incision was made along the mark and a dissection was then performed cautiously above the deep fascia from lateral to medial; the perforators and the intercostal nerves were preserved as much as possible. During the dissection, most perforators emerged near the lateral border of the rectus sheath; all of them were identified and the most robust one was chosen as the vascular pedicle. A retrograde dissection of the cutaneous perforators to the source vessel was then performed. First, a longitudinal incision was made around the perforator on the anterior sheath of the rectus abdominis. Then, the underlying rectus was split in the direction of its fibers to reduce injury. An intramuscular separation was performed retrogradely along the perforator up to where the trunk of the deep inferior epigastric artery was met. An anterogradely dissection towards the distal end along the trunk was conducted after identification of branches or terminal branches that were nourishing the rectus. The excision of the anterior sheath and the rectus was conducted in accordance with the point where the vessels entered the muscle.

#### *Flap Elevation*

**Fig. 1:**
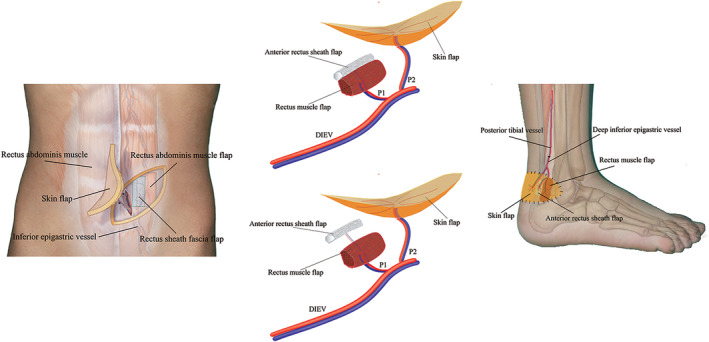
Schematic diagram. (Left) Donor site. (Middle upper) The muscle flap was attached to the fascia flap. (Middle lower) The muscle flap was separated from fascia flap. (Right) Recipient site. DIEV, deep inferior epigastric vessel; p1, perforator 1, p2, perforator 2.

**Fig. 2:**
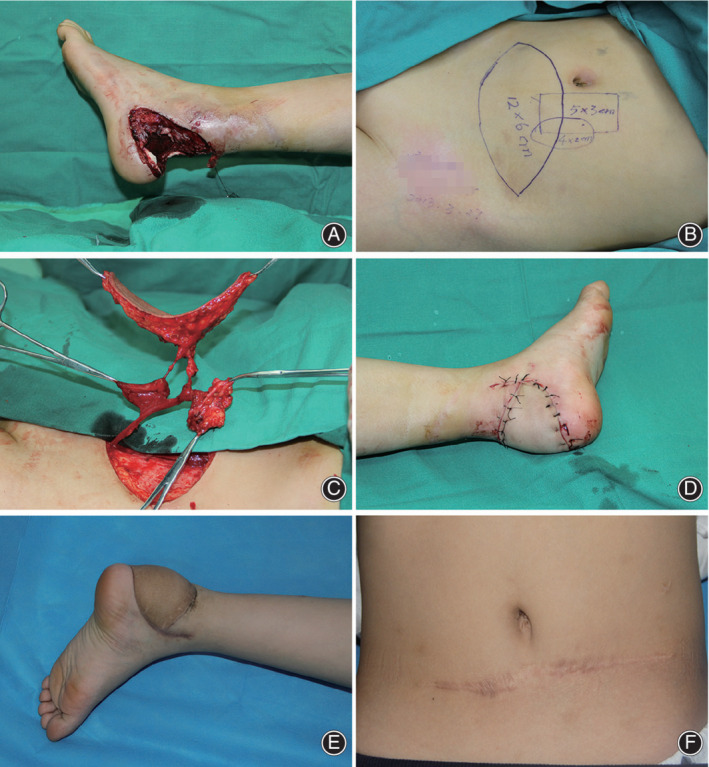
An 8‐year‐old boy presented with composite tissue defects in the left heel as a result of a motorcycle spoke injury. (A) After thorough debridement, a 12 × 6 cm^2^ skin defect, 4‐cm Achilles tendon damage, and a dead space were left. (B) To reconstruct the defects, we designed a free chimeric deep inferior epigastric artery perforator (DIEP) flap according to the size and three‐dimensional features of defects. (C) After harvest and prefabrication, (D) the flap was transferred to the left heel, with several stitches in the skin paddle to secure temporarily. Revascularization of the flap was then attained through end‐to‐end anastomoses to the posterior tibial artery and concomitant veins. After that, the broken ends of the Achilles tendon were bridged with the anterior sheath of the rectus and the dead space was filled with rectus abdominis block. The donor site was initially closed. The flaps survived well without complications and the wound healed within 2 weeks. (E) One year postoperatively, the patient had restored full ankle mobility and (F) a slightly noticeable linear scar was left on the abdominal wall.

**Fig. 3:**
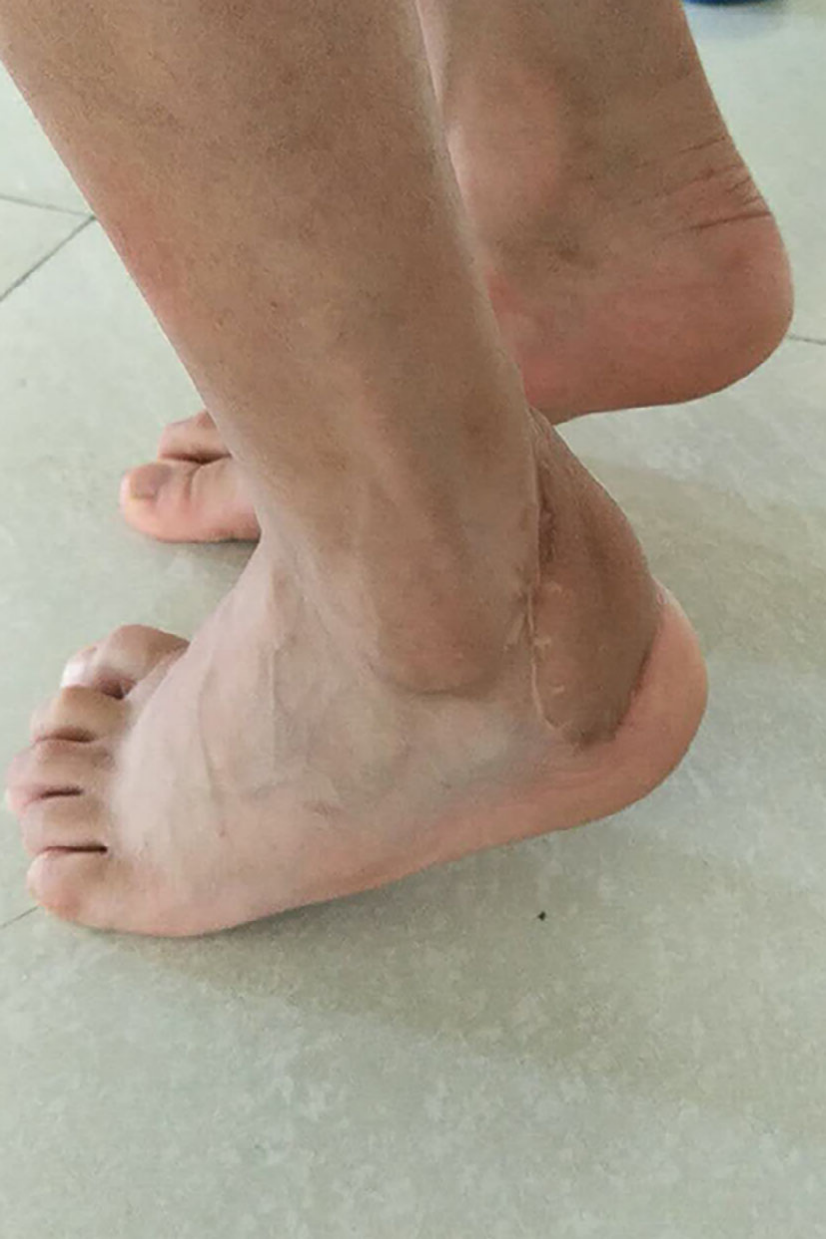
At the 2‐year follow‐up, the flap was slightly thickened. The extension and flexion of the ankle joint were normal and the patient had restored normal gait.

**Fig. 4:**
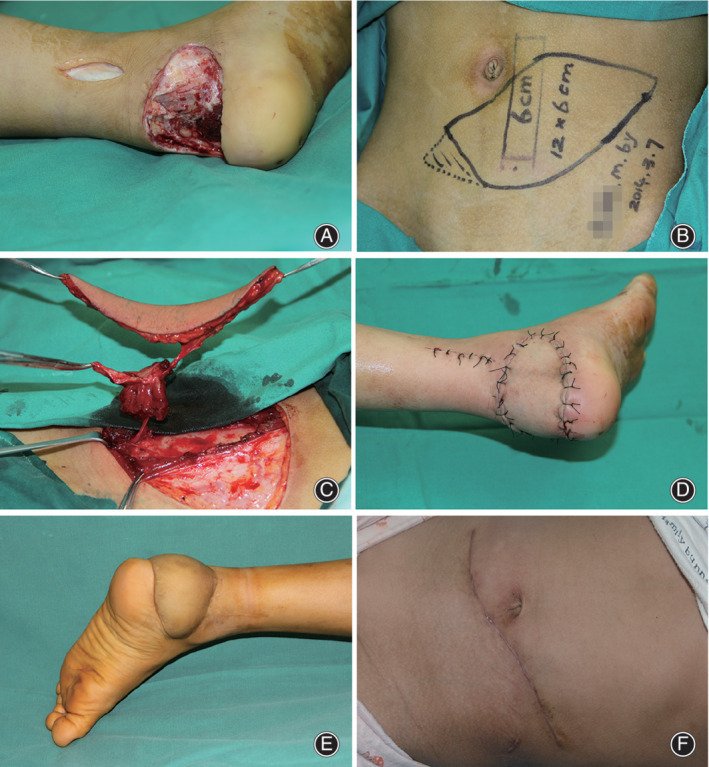
A 6‐year‐old boy suffered from a motorcycle spoke injury with composite tissue defects of the skin and soft tissue in the right heel. Emergent surgery was performed. (A) Following extensive debridement, a 12 × 6 cm^2^ skin defect was present, with 6‐cm damage of the Achilles tendon and a large dead space. (B) A chimeric deep inferior epigastric artery perforator (DIEP) flap was designed. (C–D) After harvesting and transferring the flap, the posterior tibial artery and a concomitant vein were used as recipient vessels. The donor site was initially closed, and the postoperative course was uneventful. (E–F) Two years postoperatively, the aesthetic results were satisfactory in both the donor and recipient sites.

**Fig. 5:**
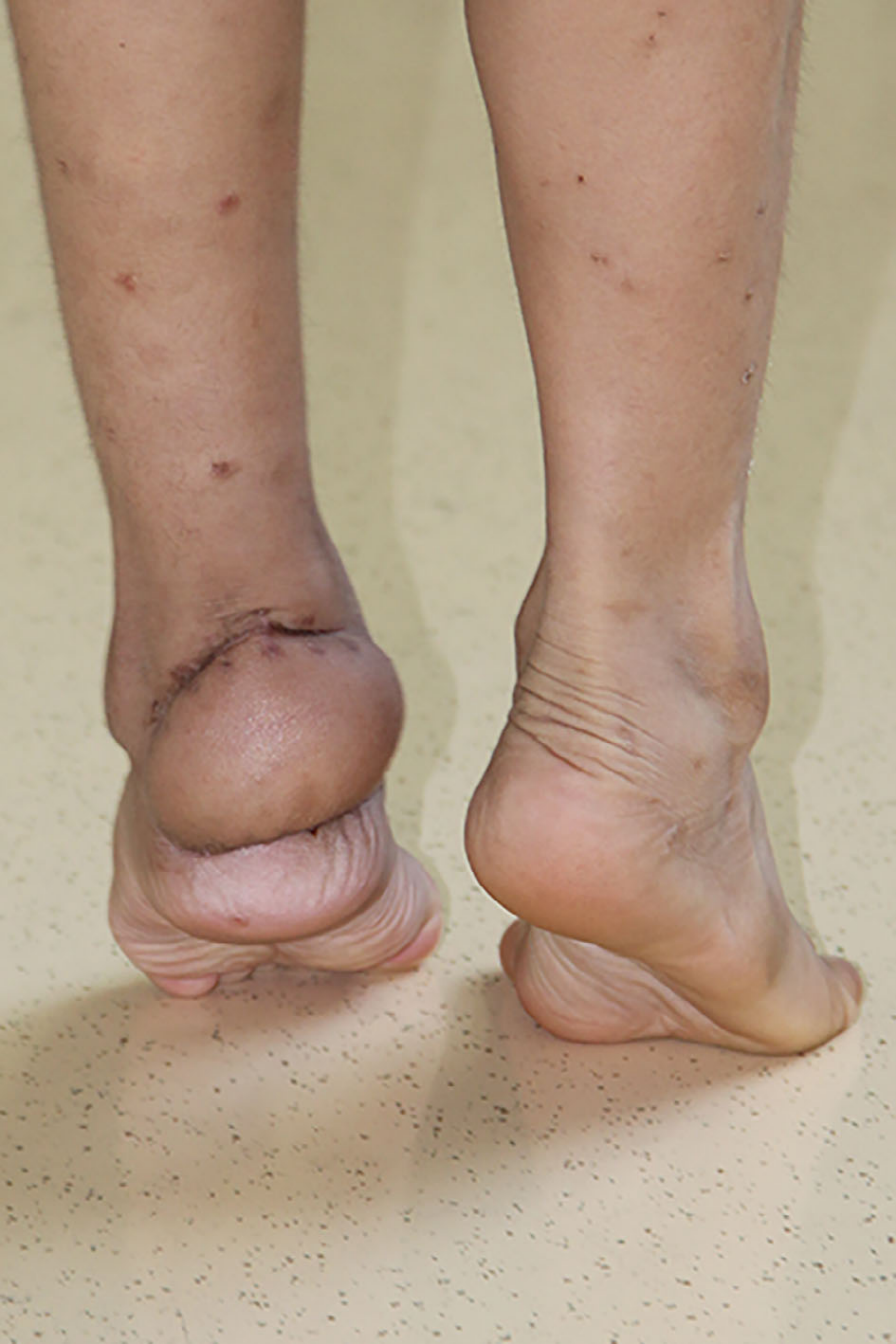
At the 3‐year follow‐up, good ankle function was accomplished, and the gait was normal. The flap had a good contour without signs of obvious hyperpigmentation.

**Fig. 6:**
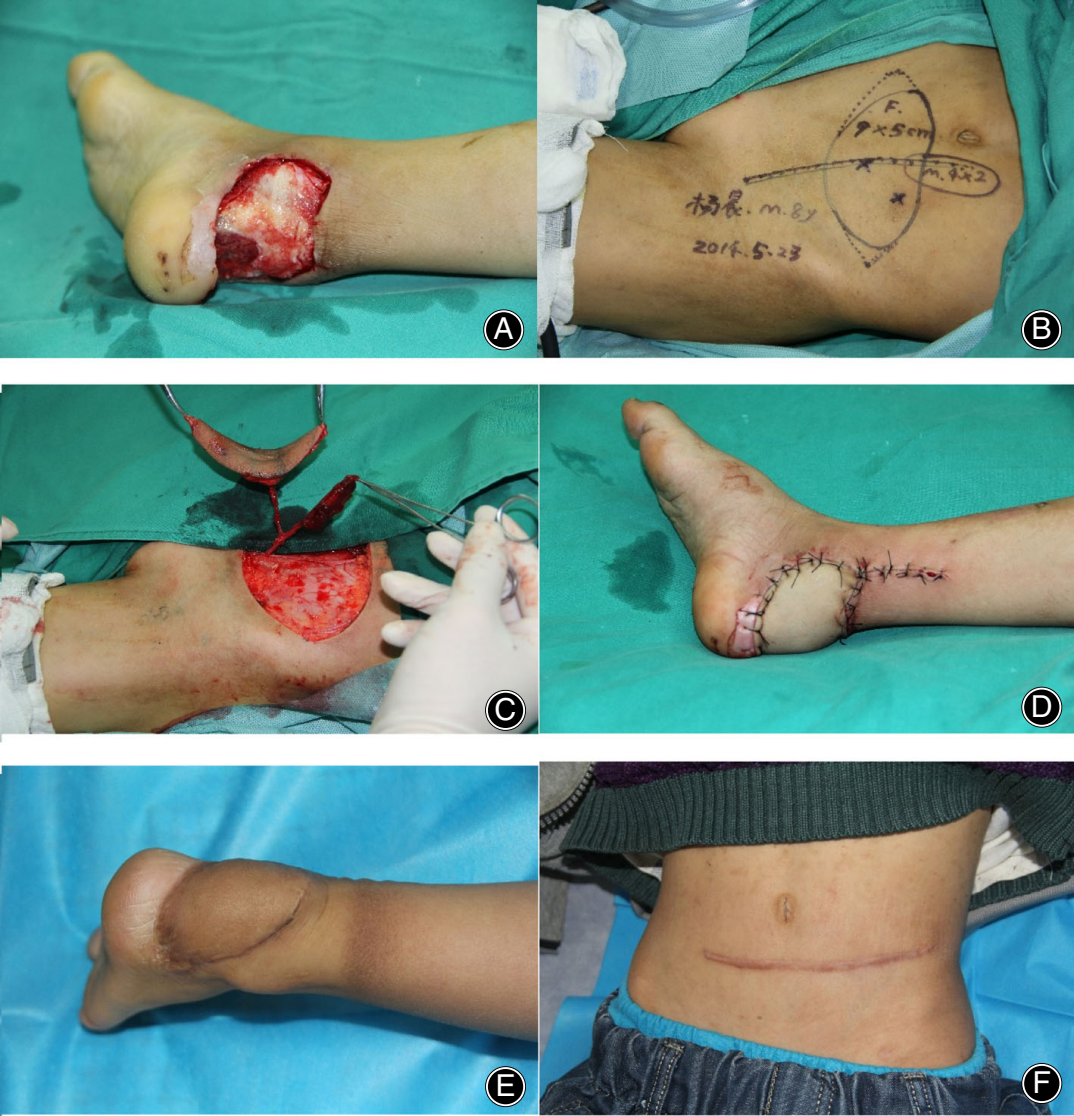
A 8‐year‐old boy suffered from Achilles tendon rupture and skin and soft tissue defect due to Motorcycle spoke injury. He underwent debridement, Achilles tendon repair and dressing change in the local hospital and was transferred to our center for further treatment 9 days later. (A) After thorough debridement, A 12×6 cm^2^ skin defect and 3cm Achilles tendon defect with a 5×3 dead space underneath were left. (B–D) A chimeric DIEP flap was designed, harvested and transferred, its pedicle was anastomosed with posterior tibial artery and a concomitant vein. The initial donor site closure was accomplished. (E–F) 2 years postoperatively, the aesthetic results were satisfactory both the donor and recipient sites.

The anterior sheath was excised at a length equal to the Achilles tendon defect and was at least 2.5 times as wide as the transvers diameter of the Achilles tendon. On this basis, two types of fascial‐muscular flap were designed according to the perforator position (Fig. 1). In type 1, the muscle flap was separated from the fascia flap (Figs 2, 3, 4, 5); in type 2, the muscle flap was attached to the fascia flap without perforator dissection from the fascia flap (Fig. 6). To confirm adequate blood supply, the infusion of each component was checked independently and a proximal dissociation along the trunk was conducted to harvest the required length of pedicle.

#### *Flap Transfer and Defects Repair*

Subsequently, the chimeric DIEP flap was transferred to the recipient site, and the deep inferior epigastric artery was then end‐to‐end anastomosed to the recipient artery with 10/0 or 11/0 nylon sutures. The anterior sheath was prefabricated by folding it in half and seaming the side with continuous stitches. Then, the sheath was grafted to bridge the tendon ends with Kessler's sutures. The muscle mass was then inset to fill the dead cavity and was fixed with several interrupted sutures. Finally, the skin flap was used to cover the skin defect. A dose of 100 mL of dextran‐40 was administered intravenously during the operation. Using this chimeric technique, each part of the flap could be inserted accurately into the defects tension‐free with maximum degree of freedom, which was guaranteed by long vessels between each part of the flap.

#### *Donor Site Closure and Postoperative Administration*

The abdominoplasty was performed with interrupted sutures in the rectus and continuous stitches in the anterior sheath, as well as layer‐to‐layer closure of the skin incision.

Postoperatively, the drains were removed on day 3. Antibiotics were used for 3 days. Intravenous dextran‐40 was administered: 100–200 mL/day for 5–7 days.

## Results

A total of six reconstructions were performed in the series by the same senior author. The area of the skin paddle ranged from 10 × 4 cm^2^ to 14 × 7 cm^2^, the length of the anterior sheath of the rectus ranged from 3 to 6 cm, and the size of the muscle mass ranged from 4 × 3 cm^2^ to 6 × 4 cm^2^. Primary donor site closure was accomplished in all patients. Among these cases, five chimeric DIEP flaps survived well, without infection or other complications. The remaining one patient underwent partial necrosis of the skin paddle caused by compression of venous outflow due to foot swelling, which was healed after the removal of several stitches and routine dressing changes.

After an average follow‐up of 18.6 months (range, 10–36 months), five patients were satisfied with the surgical results; one child needed secondary defatting surgery for wearing shoes. The flaps showed good texture match and contour, with only a slightly noticeable linear scar on the abdominal wall of each patient. No abdominal hernia, bulging, or impaired muscle movement were observed. The donor‐site morbidity was insignificant, with minimal functional impairment. Compared to the unaffected side, the affected side showed no obvious difference regarding to the ankle movement, tiptoe function and patient gait during the follow‐up period. Good ankle function was observed in all patients. Three case reports are shown in Figs 2, 4, and 6.

## Discussion

The composite soft‐tissue defects in children, including mass skin loss, Achilles tendon defects, and dead space, are among the most challenging tasks in plastic surgery^1^. The small vessels and the tendency for vasospasm pose unique difficulties in anastomoses^14^, ^15^. The lack of spare tissue around the heel and the poor blood perfusion in extremities also created a huge obstacle for reconstruction. In addition, the limited impacts on future growth and development should be taken into consideration.

### *Local Flaps for Heel Repair*

A variety of local flaps are reported to be effective in reconstruction of the heel after motorcycle spoke injuries, including the saphenous neurocutaneous flap, the posterior tibial perforator flap, the sural neurocutaneous flap, the peroneal artery perforator flap, and the sliding gastrocnemius musculocutaneous flap^5^. The limitations of regional flaps are obvious. First, insufficient surface area around the heel makes these flaps less applicable, because skin grafts are always required due to failure to close the donor wound initially, thus leaving an unsightly and itchy second donor site behind. Second, in the present study the regional flaps included a small amount of tendinous and subcutaneous tissues, and, thus, were not capable of bridging the large defects of the Achilles tendon and filling the dead cavity.

### *Free Anterolateral Thigh Perforator Flap and Circumflex Scapular Artery Perforator Flaps for Heel Repair*

With the development of microsurgical techniques, free flap transplantation has become the preferred method of treatment for children in many centers. The success rate of free flaps is similar in children and adults^1^, ^16^, ^17^. To sum up, various forms of flaps with different tissues in different donor areas have pushed microscopic reconstruction in children to a new level, enabling more accurate repair and low incidence of complications.

The anterolateral thigh perforator flap (ALT) is a multipurpose flap, which is often used in the repair of children's lower limbs^2^, ^18^, ^19^, ^20^. However, in children, due to growth and development of children, the head and trunk area is relatively large, while the limb area is small, so the flap area is limited. Based on Cao *et al*. (2019), the ALT is, therefore, suitable for moderate skin loss (less than 120 cm^2^) in heels^21^. In addition, the dense and thick skin and subcutaneous tissues of ALT restrict the ability to cover the heel, where elasticity, flexibility, and thinness are required. Circumflex scapular artery perforator (CSAP) flap reconstruction is another popular treatment, which was first reported in 2007^22^, ^23^, ^24^. However, without tendinous tissue present in this flap, the second donor site is required to reconstruct Achilles tendon defects, thus reducing its scope for application.

### *Chimeric Deep Inferior Epigastric Artery Perforator Flap for Heel Repair*

The DIEP flap was first reported by Koshima in 1989^12^, ^25^. Based on the traditional transverse rectus abdominis myocutaneous (TRAM) flap, the DIEP flap was elevated from the muscle layer and only supported by deep inferior epigastric perforators. The new form of flap attenuated the abdominal wall after flap harvest by saving the abdominal muscles. Currently, DIEP flap is widely used and is considered the most reliable option for autologous breast reconstruction after mastectomy, due to the large amount of skin and soft tissue available and low incidence of donor site complications^14^, ^26^, ^27^, ^28^, ^29^. The application of the DIEP flap in the head, neck, limbs, and other body parts has also achieved good results^30^, ^31^, ^32^, ^33^, ^34^, ^35^, ^36^. Repairing the dead space of the heel can be challenging because ineffective closure can lead to seroma or hematoma and increase the risk of bacterial infection^37^, ^38^. Vascularized masses of soft tissue, including fascia and muscles, are effective fillers to eliminate dead space and have a strong ability to resist infection. To bridge the thick, strong Achilles tendon, more than one tendon is usually needed. With few spare tendons across the body, this is a real problem. Prefabricated aponeurosis, including fascia lata and the anterior sheath of the rectus abdominis, is another solution. With accessibility to the large skin flap for wound resurfacing, the anterior sheath of the rectus abdominis for Achilles tendon bridging, and the muscle block for dead space filling, the chimeric DIEP flap has great potential in pediatric heel reconstruction. The composite defects include various sorts of tissue losses and are three‐dimensional, with each part lying in different layers. Given this, apart from including the diverse tissues, the flap should be of freedom to configure and place each component without tether or tension. The traditional musculocutaneous flaps and composite flaps can only be used for simple two‐dimensional repair due to the lack of mobility caused by the close connection of skin, fascia, and muscle components. In addition, the flap does not carry tendon tissue and cannot be used to reconstruct the Achilles tendon. The composite flap needs anastomosis of multiple blood vessels, which is difficult to achieve when recipient vessels are limited. Recently, several pioneering works have been reported, in which the perforator‐based chimeric flaps were applied for economical tissue use^8^, ^9^, ^10^, ^11^, ^39^, ^40^, ^41^, ^42^, ^43^. The chimeric flap has many advantages over other compound flaps, including conjoined and composite flaps. The former has various tissues with maximum mobilities between each element and minimum vessel requirements at the recipient site.

Based on previous works, in the present study we introduce a chimeric DIEP flap for three‐dimensional heel reconstruction in children.

### *Technical Essentials for Flap Revascularization*

According to previous studies^1^, ^21^, ^44^, one robust perforator with a proper caliber is sufficient to support a skin flap as large as 24 × 8 cm^2^; one to two perforators were enough for the skin paddle. The outflow of veins was vulnerable because of the thin vessel wall and slower flow. One case in our series with remote partial necrosis might be attributed to venous congestion. Therefore, we suggest retaining the superficial epigastric vein for backup to reinforce the drainage and paying great attention to vein anastomosis. The dissection of perforators and the distal trace of the DIEA is the key to harvesting the chimeric flap. With the assistance of an operating microscope, after the elevation of the skin paddle from lateral to medial above the deep fascia, the retrograde dissection was performed along the robust perforator or perforators until the trunk of the DIEA was reached, before the separation towards the distal end of the DIEA. After identification of branches or terminal branches that were nourishing the rectus and fascia, the excision of the anterior sheath and the rectus was conducted in accordance with the point where the vessels entered the muscle. Depending on the anatomical relation between the facial vessels and the muscular vessels, two types of facia‐muscular flaps could be harvested; each of them is of sufficient degrees of freedom for insert. When placing the flap, each element should be handled carefully; any tethering or twisting could result in necrosis and loss. In our experience, skin flaps longer than 12 cm should be positioned in oblique orientation to ensure adequate blood supply^1^, ^21^, ^44^. The oblique cutaneous flap, which lay in zones I, II, and III, referring to the angiosome anatomy, could avert remote necrosis. The revascularization of the whole three elements in the DIEP flap is achieved through one set of anastomoses between DIE vessels and posterior tibial vessels in an end‐to‐end manner.

### *The Technical Essentials for Dead Space Elimination*

High‐energy trauma, such as motorcycle spoke injury, imposed on the heel often results in vascular lesions, soft tissue loss, and devitalization of adjacent tissues. Thorough debridement is essential to avoid bacterial infection. However, the radical debridement itself creates a dead space in the depth of the wound, which may cause spread of infection and resultant loss of larger tissues if not filled adequately. The muscular flap is considered a stronger anti‐infective, vascularized tissue than other flaps like the de‐epithelialized skin flap because it has robust blood supply^45^, ^46^, ^47^, ^48^, ^49^, ^50^, ^51^. With the aim of dissecting the DIEA towards the distal end and including as many muscular branches as possible, the rectus muscle mass in our chimeric DIEP flap served well as no infection was found in our case series. Hernias and bulges did not occur within the follow‐up period. However, further analysis of the long‐term effects is needed, especially in the situation where a female patient becomes pregnancy.

### *Technical Essentials for the Achilles Tendon Defect Repair*

A prefabricated anterior sheath of the rectus abdominis which matched the dimension of the Achilles tendon defect was used in the chimeric DIEP flap. After the identification of muscular branches or terminal vessels of DIEA, the dissociation of the anterior sheath commenced. The fascia was excised at least 2.5 times as wide as transverse diameter of Achilles tendon, then folding in half and seamed with continuous stitches on the sides. With long pedicles at both the DIEA cut end and the skin paddle, the bridging should be easily accessible without causing disturbance. To reduce the morbidities after sheath and muscle harvest, the excision must be parallel with the fiber direction of the rectus and limited to the lateral part of muscle. Furthermore, some scholars believe that damage to the intercostal nerve during flap elevation is the main reason for abdominal morbidity rather than damage to the muscle and its fascia^52^, ^53^, ^54^, ^55^. Therefore, the intercostal motor nerve, especially the dominant nerve, should be preserved as much as possible^55^.

The final goal of the reconstruction in children is to achieve adequate functional and morphological rehabilitation of the treated area at the lowest possible price. Hence, tissue type, matching shape, and volume of the surgical defect should be taken into consideration, as well as minimal compromise at the donor site. Despite the proven versatility of the chimeric DIEP, the drawbacks are also obvious. The fat hypertrophy was the main problem. We have noticed that many DIEP flaps, either in this case series or others that we have studied of different design, gain various degrees of bulkiness when patients grow and gain weight. Some of them need further defatting surgery for wearing shoes. The inability to reconstruct cutaneous sensation is another drawback of the DIEP flap. In addition, the combination of multiple techniques requires high levels of skill and specialized instruments. This should be kept in mind when future modifications are developed.

In conclusion, the chimeric DIEP flap is a rational design for three‐dimensional heel reconstruction in children.
